# Supramolecular Porphyrin Nanostructures Based on Coordination-Driven Self-Assembly and Their Visible Light Catalytic Degradation of Methylene Blue Dye

**DOI:** 10.3390/nano10112314

**Published:** 2020-11-22

**Authors:** Nirmal Kumar Shee, Min Kyoung Kim, Hee-Joon Kim

**Affiliations:** Department of Applied Chemistry, Kumoh National Institute of Technology, Gumi 39177, Korea; nirmalshee@gmail.com (N.K.S.); min1107min@naver.com (M.K.K.)

**Keywords:** porphyrin triad, metal-ligand coordination, nanostructure, photocatalytic degradation of dye

## Abstract

A series of porphyrin triads (**1**–**4**), in which each triad is composed of a Sn(IV) porphyrin and two free-base (or Zn(II)) porphyrins, was synthesized and their self-assembled nanostructures were studied. Depending on the substituent on porphyrin moieties, each triad was self-assembled into a different nanostructure. In particular, the cooperative coordination of 3-pyridyl groups in the Sn(IV) porphyrin with the axial Zn(II) porphyrins in triad **4** leads to forming uniform nanofibers with an average width of 10–22 nm. Other triads without the coordinating interaction between the central Sn(IV) porphyrin and the axial porphyrins formed irregularly shaped aggregates in contrast. The morphologies of nanofiber changed drastically upon the addition of pyrrolidine, in which pyrrolidine molecules break down the self-assembly process by coordinating with the axial Zn(II) porphyrins. All porphyrin aggregates exhibited efficient photocatalytic performances on the degradation of methylene blue dye under visible light irradiation. The degradation efficiencies after 2 h were observed to be between 70% and 95% for the aggregates derived from the four triads.

## 1. Introduction

The fabrication of highly ordered and diversified morphologies of nano- and micro-structured optoelectronic materials is of great contemporaneous interest, especially for their applications in catalysis, sensing, solar energy conversion, and storage [1,2,3,4,5,6]. Among the various building blocks, porphyrins are attractive components for the construction of self-assembled nanomaterials with well-defined shapes and dimensions [7,8,9,10,11,12,13,14,15]. Combination of porphyrins and other optoelectronic functional materials has been extensively studied to develop new nanohybrid systems which can intensify light-harvesting and charge-transfer functions [16,17,18,19,20]. Porphyrins and similar molecules have been long known to self-assemble and form large-scale aggregates in solution, but these structures are usually irregular and not acceptable for many technological applications. Therefore, the formation of well-defined, discrete (isolable) porphyrin nanostructures with a definite size, shape, and dimension is a challenging task. In the self-assembly of porphyrins, a variety of intermolecular non-covalent interactions (e.g., hydrogen bonding, ligand coordination, hydrophobic, π-π stacking, van der Waals forces, and electrostatic interactions) have been utilized [21,22,23,24,25,26,27,28,29,30]. In recent years, Sn(IV) porphyrins have been widely used as a building block for the construction of useful porphyrin nanostructures, including nanocomposites, nanosheets, nanotubes, or thin stripes on surfaces [31,32,33,34]. The oxophilic nature of Sn(IV) centers allows them to easily form stable six-coordinate complexes with the two trans-axial oxyanion ligands [35,36,37,38,39,40,41]. These synthetic Sn(IV) porphyrin-based nanostructures not only are of interest for solar energy technologies but also have potential applications as photonic (optoelectronic) materials in various micro- and nanoscale electronic devices [42,43,44].

**Chart 1:**
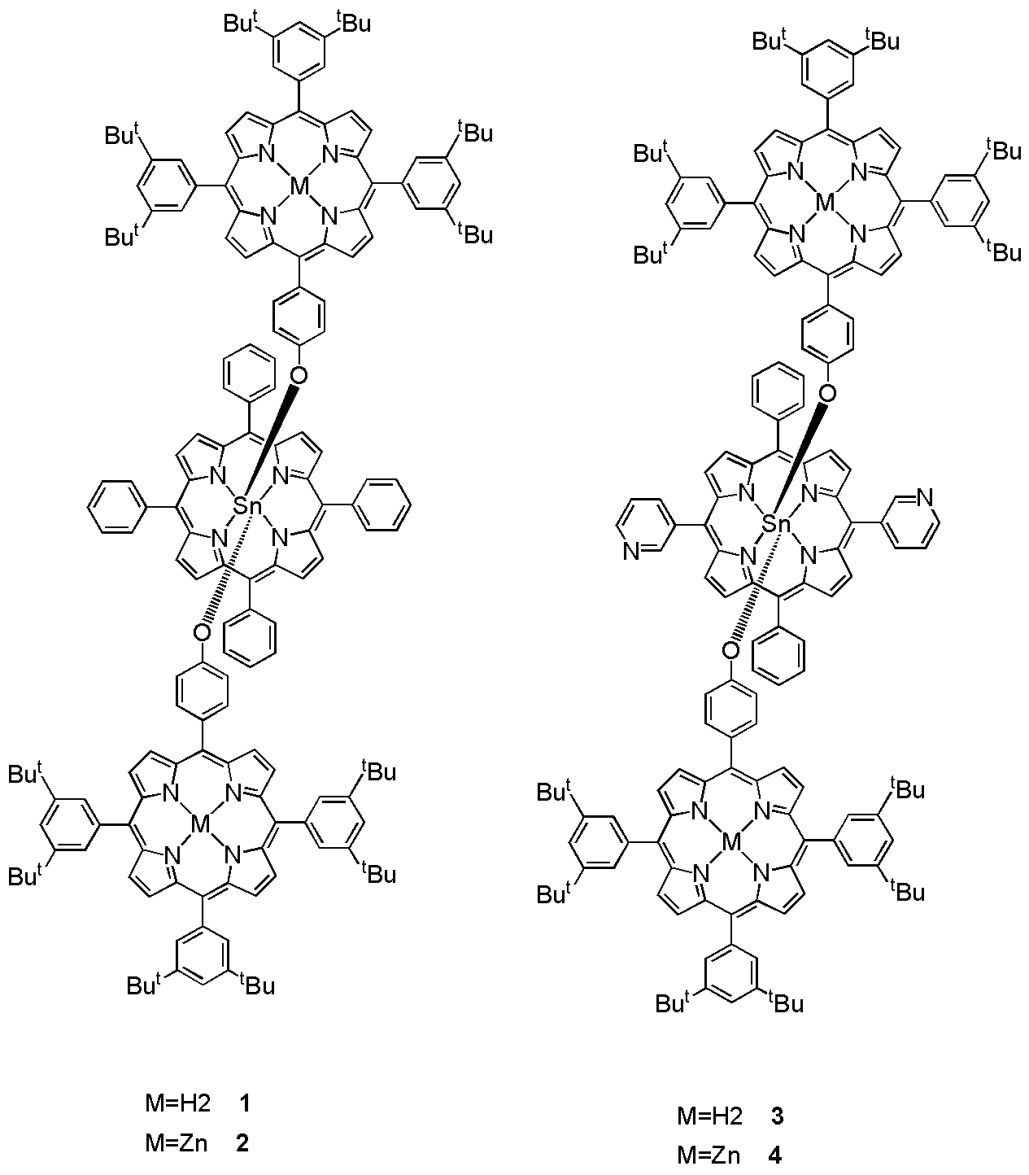
Chemical structures of triads **1**–**4**.

In this study, a series of Sn(IV) porphyrin-based triads were designed and prepared to explore their self-assembled nanostructures. We synthesized four triads using two different Sn(IV) porphyrins as well as free-base or Zn(II) porphyrins as the axial porphyrin array (Chart 1). Here, we intended to find out if the structural diversity of the triads can direct the formation of different controllable nanostructures, such as nanospheres, nanofibers, or nanorods. Furthermore, we investigated the photocatalytic performance in the degradation of organic dyes in an aqueous medium under visible light irradiation.

## 2. Materials and Methods

All chemicals were purchased from Sigma-Aldrich and used without further purification, unless otherwise noted. First, 5-(3-Pyridyl)dipyrromethane was synthesized according to a similar procedure in the literature (using 3-pyridinecarboxaldehyde instead of 4-pyridinecarboxaldehyde) [45]. Trans-dihydroxo-(5,10,15,20-tetrakis(phenyl)porphyrinato)tin(IV) was synthesized according to a procedure in the literature [42]. All experiments reported here were carried out by using standard Schlenk line techniques under a dry argon atmosphere. Toluene and pyrrole were purified by distillation from a sodium/benzophenone ketyl solution and a solution of calcium hydride, respectively. Steady-state UV-vis spectra were recorded on a Shimadzu UV-3600 spectrophotometer (Shimadzu, Tokyo, Japan). The fluorescence spectra were recorded with a Shimadzu RF-5301PC fluorescence spectrophotometer (Shimadzu, Tokyo, Japan). The ^1^H NMR spectra were obtained on a Bruker BIOSPIN/AVANCE III 400 spectrometer at 293 K (Bruker BioSpin GmbH, Silberstreifen, Rheinstetten, Germany). Electrospray ionization mass spectra (ESI-MS) mass spectra were recorded on a Thermo Finnigan Linear Ion Trap Quadrupole mass spectrometer (Thermo Fisher Scientific, Seoul, South Korea). Elemental analysis was performed by using the EA 1110 Fisons analyzer (Used Lab Machines Limited, London, England). Field emission scanning electron microscope (FE-SEM) images were obtained from MAIA III (TESCAN, Brno, Czech Republic). X-ray diffraction patterns were obtained using a SWXD X-MAX/2000-PC diffractometer (Rigaku, Tokyo, Japan) with Cu Kα radiation (*λ* = 1.5418 Å, 0.02° step^−1^). Dynamic light scattering (DLS) experiments were performed using a NanoBrook 90Plus DLS size analyzer (Brookhaven, NY, USA). The photocatalytic performances of the nanostructures were studied by the degradation of methylene blue (MB). In the typical process, 5 mg of the photocatalyst was added to 100 mL aqueous MB solution (10 mg L^−1^) and then magnetically stirred in the dark for 30 min, which allowed the nano-aggregates to reach an adsorption equilibrium. After that, the above mixture was exposed to visible light irradiation from a 150 W Xenon arc lamp (ABET technologies, Old gate lane Milford, Connecticut, USA) at room temperature. All the samples were collected by centrifugation at given time intervals to determine MB concentration by UV-vis spectroscopy at 664 nm.

### 2.1. Synthesis of meso-5-(4-hydroxyphenyl)-10,15,20-tris(3,5-di-tert-butylphenyl)porphyrin H_2_L^1^

This porphyrin was synthesized by mixed aldehyde condensation in propionic acid using 3.0 equiv. of 3,5-di-*tert*-butylbenzaldehyde, 1.0 equiv. of 4-hydroxybenzaldehyde, and 4.0 equiv. of pyrrole. Pyrrole (0.77 mL, 11.1 mmol) was added drop-wise to a solution of 4-hydroxybenzaldehyde (0.34 g, 2.8 mmol) and 3,5-di-*tert*-butylbenzaldehyde (1.82 g, 8.3 mmol) in propionic acid (60 mL) under reflux. After, the mixture was stirred for 3 h. The reaction mixture was cooled to 5 °C to precipitate the product. The solid precipitate was filtered and washed with hot water and then dried at 50 °C to furnish the crude. Then, the mixture was purified by column chromatography (SiO_2_, eluent: CH_2_Cl_2_/MeOH = 98:2) to afford the compound **H_2_L^1^**. The crude was recrystallized from CH_2_Cl_2_/hexane to give a violet purple powder. Yield: 0.134 g (5%). Anal. Calcd for C_68_H_78_N_4_O: C, 84.43; H, 8.13; N, 5.79; R, 1.65. Found: C, 84.27; H, 8.81; N, 5.70; R, 1.22. ^1^H NMR (400 MHz, CDCl_3_, ppm): *δ* −2.71 (s, 2H, NH), 1.50 (s, 54H, ^t^Bu), 7.19 (d, *J* = 8.5 Hz, 2H, H2,6-PhOH), 7.77 (s, 3H, H4-Ar), 8.08 (m, 8H, *meso*-O-Ar), 8.87 (d, *J* = 5.7 Hz, 8H, β-pyrrole). UV-visible (CHCl_3_): *λ*_nm_ (log *ε*), 418 (5.30), 517 (4.18), 551 (3.90), 593 (3.60), 650 nm (3.57). Emission (CHCl_3_, *λ*_nm_): 655, 715.

### 2.2. Synthesis of meso-[5-(4-hydroxyphenyl)-10,15,20-tris(3,5-di-tert-butylphenyl)porphyrinato] Zinc(II) ZnL^1^

Zn(OAc)_2_·2H_2_O (0.053 g, 0.29 mmol) was added to a solution of *meso*-5-(4-hydroxyphenyl)-10,15,20-tris(3,5-di-*tert*-butylphenyl)porphyrin (0.1125 g, 0.12 mmol) in a mixed solvent of tetrahydrofuran (25 mL) and in methanol (25 mL). The mixture was refluxed for 2 h. The solvent was then removed and the solid product was purified by column chromatography (SiO_2_, eluent: CH_2_Cl_2_/MeOH = 98:2). The crude solid was recrystallized from CH_2_Cl_2_/hexane to give a brownish red **ZnL^1^**. Yield: 0.096 g (80%). Anal. Calcd for C_68_H_76_N_4_OZn: C, 79.24; H, 7.43; N, 5.44; R, 7.89. Found: C, 79.07; H, 7.67; N, 5.32; R, 7.94. ^1^H NMR (400 MHz, CDCl_3_, ppm): *δ* 1.51 (s, 54H, ^t^Bu), 7.19 (d, *J* = 8.1 Hz, 2H, H2,6-PhOH), 7.79 (s, 3H, H4-Ar), 8.10 (m, 8H, *meso*-O-Ar), 8.99 (d, *J* = 5.8 Hz, 8H, β-pyrrole). UV-visible (CHCl_3_): *λ*_nm_ (log *ε*), 419 (5.30), 547 (4.04), 586 nm (3.70). Emission (CHCl_3_, *λ*_nm_): 600, 645.

### 2.3. Synthesis of Triad ***1***

A mixture of **H_2_L^1^** (0.132 g, 0.136 mmol) and trans-dihydroxo-[5,10,15,20-tetraphenylporphyrinato]tin(IV) (0.052 g, 0.068 mmol) was added to anhydrous toluene (20 mL) and refluxed for 48 h under argon atmosphere. After that, the solvent was evaporated in vacuo. Then, the resulting residue was dissolved in a minimum amount of CH_2_Cl_2_ and loaded onto a neutral alumina column. Triad **1** was eluted with CH_2_Cl_2_, after which it was recrystallized using a mixture of CHCl_3_/*n*-hexane to give reddish powder. Yield: 0.117 g (65%). Anal. Calcd for C_180_H_182_N_12_O_2_Sn: C, 81.15; H, 6.89; N, 6.31; R, 5.66. Found: C, 81.02; H, 6.99; N, 6.23; R, 5.76. ^1^H NMR (400 MHz, CDCl_3_, ppm): *δ* −2.90 (s, 4H, NH), 1.51 (s, 108H, ^t^Bu), 2.45 (d, *J* = 8.4 Hz, 4H, α-bridging phenyl), 6.52 (d, *J* = 8.4 Hz, 4H, β-bridging phenyl), 7.80 (m, 18H, *m*,*p*-phenyl{central} + H4-Ar {axial}), 8.02 (m, 12H, *meso*-O-Ar-axial), 8.30 (d, *J* = 7.6 Hz, 8H, *meso*-O-phenyl-central), 8.45 (d, *J* = 8.2 Hz, 4H, β-pyrrole-axial), 8.55 (d, *J* = 8.2 Hz, 4H, β-pyrrole-axial), 8.80 (s, 8H, β-pyrrole-axial), 9.19 (s, 8H, β-pyrrole-Sn). UV-visible (CHCl_3_): *λ*_nm_ (log *ε*), 419 (5.81), 518 (4.59), 558 (4.15), 602 nm (4.08). Emission (CHCl_3_, *λ*_nm_): 652, 720.

### 2.4. Synthesis of Triad ***2***

A mixture of **ZnL^1^** (0.14 g, 0.136 mmol) and trans-dihydroxo-[5,10,15,20-tetrakis(phenyl)porphyrinato]tin(IV) (0.052 g, 0.068 mmol) was added to anhydrous toluene (20 mL) and refluxed for 48 h under argon atmosphere. Then, the reaction mixture was cooled to room temperature, 20 mL of *n*-pentane was added, and it was stirred for 2 h. The reddish precipitate was filtered and dried in vacuo. Yield: 0.133 g (70%). Anal. Calcd for C_180_H_178_N_12_O_2_SnZn_2_: C, 77.46; H, 6.43; N, 6.02; R, 10.09. Found: C, 77.32; H, 6.74; N, 5.98; R, 9.96. ^1^H NMR (400 MHz, CDCl_3_, ppm): *δ* 1.50 (s, 108H, ^t^Bu), 2.57 (d, *J* = 8.4 Hz, 4H, α-bridging phenyl), 6.70 (d, *J* = 8.4 Hz, 4H, β-bridging phenyl), 7.78 (m, 18H, *m*,*p*-phenyl{central} + H4-Ar {axial}), 8.07 (m, 12H, *meso*-O-Ar-axial), 8.28 (d, *J* = 7.6 Hz, 8H, *meso*-O-phenyl-central), 8.45 (d, *J* = 8.2 Hz, 4H, β-pyrrole-axial), 8.55 (d, *J* = 8.2 Hz, 4H, β-pyrrole-axial), 8.85 (s, 8H, β-pyrrole-axial), 9.22 (s, 8H, β-pyrrole-Sn). UV-visible (CHCl_3_): *λ*_nm_ (log *ε*), 421 (5.78), 554 (4.48), 605 nm (4.23). Emission (CHCl_3_, *λ*_nm_): 590, 640.

### 2.5. Synthesis of 5,15-bis(3-Pyridyl)-10,20-bis(phenyl)Porphyrin H_2_L^2^

First, 5-(3-Pyridyl)dipyrromethane (0.500 g, 2.24 mmol) and benzaldehyde (0.23 mL, 2.24 mmol) were dissolved in 500 mL of anhydrous CH_2_Cl_2_ under argon atmosphere. The mixture was cooled at 5 °C with an ice bath and trifluoroacetic acid (0.5 mL, 6.5 mmol) was added drop-wise. The reaction mixture was stirred at 25 °C for 4 h and then DDQ (2,3-dichloro-5,6-dicyano-1,4-benzoquinone) (1.015 g, 4.48 mmol) was added. Then, the mixture was stirred at room temperature for 1 h. Next, 1 mL of NEt_3_ was added to quench the reaction. The mixture was then filtered through alumina and the solvent was removed by vacuum. Purification of the crude was done using column chromatography (silica, CHCl_3_/MeOH from 100/0 to 98/2) and afforded a purple solid. Recrystallization (CHCl_3_/*n*-hexane) afforded 290 mg of purple compound. Yield: 42%. Anal. Calcd for C_42_H_28_N_6_: C, 81.80; H, 4.58; N, 13.62. Found: C, 81.65; H, 4.75; N, 13.60. ^1^H NMR (400 MHz, CDCl_3_, ppm): *δ* −2.83 (s, 2H, NH), 7.75 (m, 8H, *m*,*p*-Ph + H5-Py), 8.20 (d, *J* = 6.7 Hz, 4H, phenyl), 8.52 (d, *J* = 7.7 Hz, 2H, H4-Py), 8.78 (d, *J* = 4.7 Hz, 4H, β-pyrrole), 8.89 (d, *J* = 4.7 Hz, 4H, β-pyrrole), 9.04 (d, *J* = 5.0 Hz, 2H, H6-Py), 9.44 (s, 2H, H2-Py). UV-visible (CHCl_3_): *λ*_nm_ (log *ε*), 416 (5.30), 515 (4.20), 548 (3.95), 590 (3.70), 644 nm (3.62). Emission (CHCl_3_, *λ*_nm_): 652, 720.

### 2.6. Synthesis of trans-dihydroxo-[5,15-bis(3-pyridyl)-10,20-bis(phenyl)porphyrinato]tin(IV) SnL^2^

First, 5,15-bis [3-pyridyl]-10,20-bis[phenyl]porphyrin (0.2 g, 0.32 mmol) was dissolved in 50 mL pyridine. Tin(II) chloride dihydrate (0.44 g, 1.9 mmol) was added to the above solution and refluxed for 12 h. Then, the pyridine was removed and residue was dissolved in CH_2_Cl_2_ and filtered through celite pad. Then, the solvent of the filtrate was evaporated and we dissolved it into a mixture of 25 mL solvents (20 mL of THF (tetrahydrofuran) and 5 mL of H_2_O). K_2_CO_3_ (0.65 g, 2.9 mmol) was added to the reaction mixture and refluxed for 12 h. THF was removed by reduced pressure and the mixture was cooled to 0 °C to precipitate the product. Next, we filtered the solid compound and dried it in an oven. Recrystallization (CH_2_Cl_2_/CH_3_CN) afforded 199 mg of a reddish compound. Yield: 80%. Anal. Calcd for C_42_H_28_N_6_O_2_Sn: C, 65.73; H, 3.68; N, 10.95; R, 19.64. Found: C, 65.48; H, 3.79; N, 10.60; R, 20.13. ^1^H NMR (400 MHz, CDCl_3_, ppm): *δ* −7.45 (s, 2H, Sn-OH), 7.82 (m, 8H, *m*,*p*-phenyl + H5-Py), 8.33 (d, *J* = 6.6 Hz, 4H, phenyl), 8.64 (d, *J* = 7.6 Hz, 2H, H4-Py), 9.05–9.30 (m, 10H, H6-Py + β-pyrrole), 9.55 (s, 2H, H2-Py). UV-visible (CHCl_3_): *λ*_nm_ (log *ε*), 425 (5.50), 518 (4.30), 558 (4.18), 598 nm (3.85). Emission (CHCl_3_, *λ*_nm_): 609, 656.

### 2.7. Synthesis of Triad ***3***

A mixture of **H_2_L^1^** (0.077 g, 0.08 mmol) and **SnL^2^** (0.031 g, 0.04 mmol) was added to anhydrous toluene (20 mL). Then, the reaction mixture was refluxed for 48 h under argon atmosphere. After that, the solvent was removed by rotary evaporator and the resulting residue was dissolved in a minimum amount of CH_2_Cl_2_ and loaded onto a neutral alumina column. Triad **3** was eluted with CH_2_Cl_2_, after which it was recrystallized using a mixture of CHCl_3_/*n*-hexane to give a reddish powder. Yield: (0.11 g, 80%). Anal. Calcd for C_178_H_180_N_14_O_2_Sn: C, 80.19; H, 6.80; N, 7.35; R, 5.65. Found: C, 80.02; H, 6.97; N, 7.30; R, 5.71. ^1^H NMR (400 MHz, CDCl_3_, ppm): *δ* −2.91 (s, 2H, NH), 1.50 (s, 108H, ^t^Bu), 2.52 (d, *J* = 8.4 Hz, 4H, α-bridging phenyl), 6.56 (d, *J* = 8.4 Hz, 4H, β-bridging phenyl), 7.72–7.89 (m, 14H, {*m*,*p*-phenyl + H5-Py}-central + H4-Ar-axial), 8.08 (m, 12H, *meso*-O-Ar-axial), 8.28–8.37 (m, 6H, {H4-Py + *meso*-O-phenyl}-central), 8.48 (d, *J* = 8.2 Hz, 4H, β-pyrrole-axial), 8.57 (d, *J* = 8.2 Hz, 4H, β-pyrrole-axial), 8.85 (s, 8H, β-pyrrole-axial), 9.08 (d, *J* = 8.2 Hz, 4H, β-pyrrole-central), 9.17 (d, *J* = 8.2 Hz, 4H, β-pyrrole-central), 9.27 (d, *J* = 5.0 Hz, 2H, H6-Py), 9.55 (s, 2H, H2-Py). UV-visible (CHCl_3_): *λ*_nm_ (log *ε*), 422 (5.83), 517 (4.58), 557 (4.11), 602 (4.08), 650 nm (3.85). Emission (CHCl_3_, *λ*_nm_): 652, 712.

### 2.8. Synthesis of Triad ***4***

A mixture of **ZnL^1^** (0.082 g, 0.08 mmol) and **SnL^2^** (0.031 g, 0.04 mmol) was added to anhydrous toluene (20 mL) and refluxed for 48 h under argon atmosphere. Then, the reaction mixture was cooled to room temperature and we then filtered the green precipitate. After that, it was washed with toluene and dried under vacuo. Yield: (0.096 g, 85%). Anal. Calcd for C_178_H_176_N_14_O_2_SnZn_2_: C, 76.55; H, 6.35; N, 7.02; R, 10.08. Found: C, 76.17; H, 6.82; N, 7.14; R, 9.87. ^1^H NMR (400 MHz, CDCl_3_, ppm) in the presence of 2 eq. pyridine-d_5_: *δ* 1.50 (s, 108H, ^t^Bu), 2.62 (d, *J* = 8.4 Hz, 4H, α-bridging phenyl), 6.71 (d, *J* = 8.4 Hz, 4H, β-bridging phenyl), 7.61–7.72 (m, 14H, {*m*,*p*-phenyl + H5-Py}-central + H4-Ar-axial), 7.96 (m, 12H, *meso*-O-Ar-axial), 8.10 (m, 6H, {H4-Py + *meso*-O-phenyl}-central), 8.42 (d, *J* = 8.2 Hz, 4H, β-pyrrole-axial), 8.61 (d, *J* = 8.2 Hz, 4H, β-pyrrole-axial), 8.80 (s, 8H, β-pyrrole-axial), 9.10 (d, *J* = 8.2 Hz, 4H, β-pyrrole-central), 9.20 (d, *J* = 8.2 Hz, 4H, β-pyrrole-central), 9.42 (d, *J* = 5.0 Hz, 2H, H6-Py), 9.70 (s, 2H, H2-Py). UV-visible (CHCl_3_): *λ*_nm_ (log *ε*), 455 (5.65), 572 (5.20), 614 nm (5.19). Emission (CHCl_3_, *λ*_nm_): 590, 640.

## 3. Results and Discussion

### 3.1. Syntheses

For the construction of the desired triads, we employed an “axial bonding” approach here for the design and synthesis of Sn(IV) porphyrin-based arrays having diverse structures and functions [46,47,48].

**Scheme 1:**
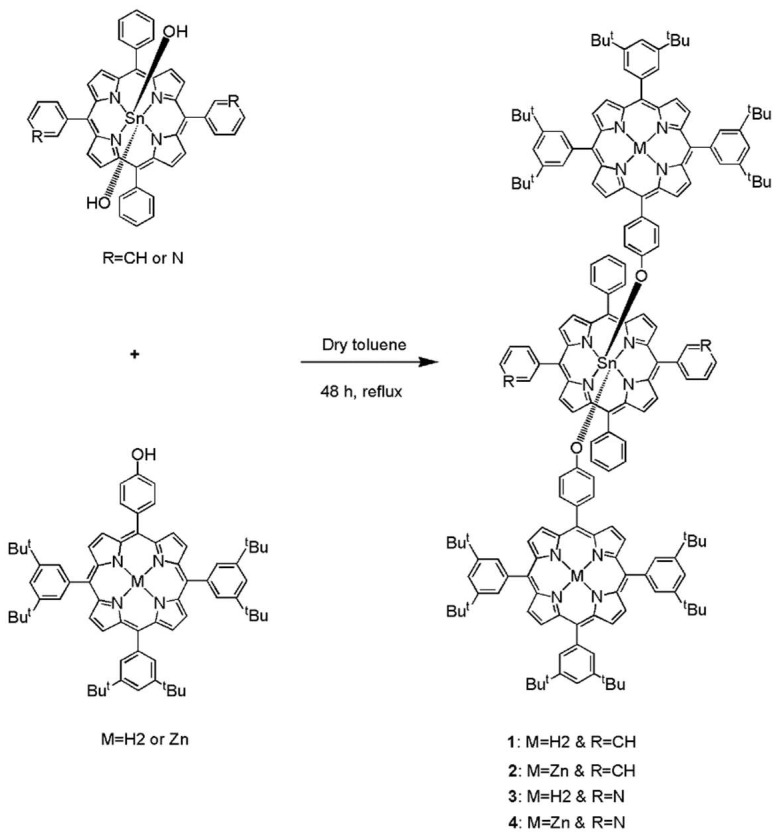
Synthesis scheme for triads **1**–**4**.

The strong tendency of Sn(IV) porphyrin to coordinate with carboxylates and aryloxides was the driving force for the formation of stable coordination arrays [46,47,48,49,50]. As illustrated in Scheme 1, all four triads were synthesized by refluxing corresponding free mono-hydroxyphenyl porphyrin or its Zn(II) complexes with trans-dihydroxo Sn(IV) porphyrins in anhydrous toluene for 48 h under an argon atmosphere. After that, the solvent was evaporated under reduced pressure and the resulting product was purified by basic alumina column chromatography using CH_2_Cl_2_ as an eluent (except triad 4). The desired product was recrystallized using a CHCl_3_/*n*-hexane mixture to afford ~ in 65–85% yield. All the synthesized compounds were fully characterized by various methods, including elemental analysis, ^1^H NMR, ESI-MS, UV-vis spectroscopy, fluorescence spectroscopy, and scanning electron microscopy.

### 3.2. Spectroscopic Characterization

The ^1^H NMR spectra of all four diamagnetic triads are shown in the supplementary information (Figures S1–S8). In addition, those of the individual unconnected monomers are also reported. As seen from the ^1^H NMR spectra of the four triads, the resonance positions of the protons on the basal Sn(IV) porphyrin components (β-pyrrolic or aromatic protons) are more or less similar to those of [Sn(OH)_2_TPP or Sn(OH)_2_DPyDPP]. On the other hand, the resonance positions, as well as the splitting patterns of the protons on their free-base axial porphyrins and its Zn(II) complexes, were quite different from those of **H_2_L^1^** or **ZnL^1^** as a result of the ring current effect influence by the Sn(IV) porphyrins. Furthermore, the ortho and meta protons (with respect to the axial ‘oxy’ group) of the bound porphyrin-aryloxy ligand in triad **1** and triad **3** were expected to experience a strong shielding effect from the ring current effect by the Sn(IV) porphyrins. The aryloxy protons appear in monomeric porphyrin **H_2_L^1^** or **ZnL^1^** at ~8.10 and 7.19 ppm as a doublet, respectively. These protons were resonated as two doublets at 2.45–2.52 and 6.52–6.56 ppm, respectively. The Δ*δ* values (i.e., *δ*(**H_2_L^1^**) − *δ*(triad **1** or triad **3**)) for these protons were ~4.7 and ~1.56 ppm, respectively. Similarly, the corresponding resonances in triad **2** and triad **4** were 2.57–2.62 and 6.70–6.71 ppm, respectively. The Δ*δ* values (i.e., *δ*(**ZnL^1^**) − *δ*(triad **2** or triad **4**)) for these protons were ~4.60 and ~1.40 ppm, respectively. The ^1^H NMR resonance pattern and magnitude of shielding observed for the β–pyrrole protons of the axial porphyrins unit attached with the Sn(IV) porphyrin moieties. The signals for the β–pyrrole protons of the free-base axial porphyrin of triad **1** or triad **3** were seen to be shifted to the upfield zone (collate to those on **H_2_L^1^**) and cleaved into a singlet at *δ* 8.80–8.85 (8H) and a pair of doublets at *δ* 8.55 (4H) and 8.45 (4H), respectively. Similarly, the corresponding resonances in triad **2** and triad **4** were 8.80–8.85 (8H), 8.57–8.61 (4H), and 8.42–8.48 (4H) ppm, respectively. Another consequence is that the resonances due to the inner imino protons of the free-base axial porphyrins are also shielded and appear at −2.90 ppm (s, 4H) in triad **1** and **3**, as compared to the corresponding protons of **H_2_L^1^** which resonate at −2.71 ppm (s, 4H)**.** Thus, the protons on the aryloxy bridges and the imino protons, at the same time, experience the shielding effect of the Sn(IV)porphyrins and the de-shielding effect of the axial porphyrins. Therefore, the ^1^H NMR method has been widely used to investigate the “axial bonding”-type architecture of similar kinds of triads [40,41]. It accounted for the interaction of the Sn(IV) porphyrins with the protons of the axial porphyrins and shows representative ring current-induced shifts and corresponding coupling resonance patterns.

Electrospray ionization mass spectra (ESI-MS) of all the four triads are shown in the supplementary information (Figures S9–S12). Although we clearly observed parent peaks corresponding to [Sn(por)(OH)_2_(H)]^+^, molecular ion peaks in all the cases were observed with very small intensities. It is likely that all the compounds were mostly fragmented during the mass spectrometry experiments. 

**Figure 1:**
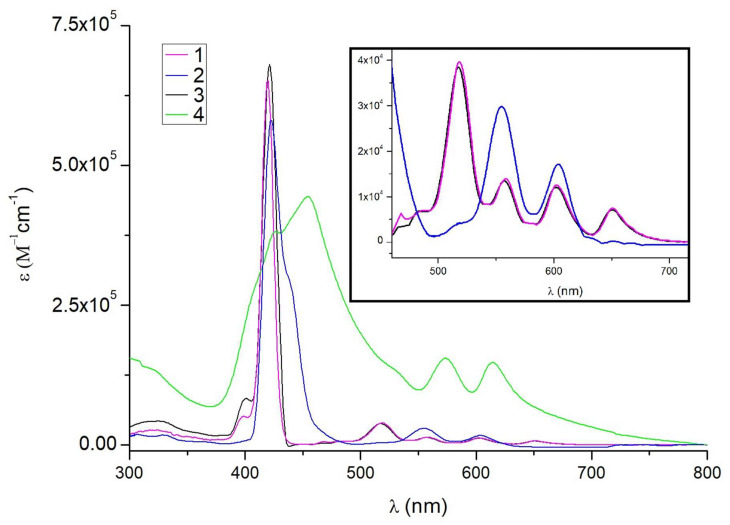
UV-visible spectra of triads **1**–**4** in CHCl_3_ (Inset: high resolution of Q band).

The UV-visible spectra of all of the four triads in CHCl_3_ solution are compared in Figure 1. The peak positions with the maximum absorbance (*λ*_max_) and molar extinction coefficient (*ε*) values for each investigated compound are reported in the experimental section. Upon inspection of Figure 1, it is clear that those spectra obtained for triads **1** and **3** are close to each other. The molar extinction coefficient values of these spectra are very much close to the sum of the monomeric porphyrin units constituting them. The *λ*_max_ values (420, 518, 557, 600, and 650 nm) of these triads are in the same ranges as those of the corresponding monomers. Insignificant variations were observed in the spectral features of these triads with respect to those of the analogous monomers that can be attributed to the substituent effects on the Sn(IV) porphyrins.

Similarly, the UV/Vis absorption data of triad **3** are indistinguishable to the corresponding data obtained for the physical mixtures of the basic monomeric porphyrins. The spectrum for **3** experienced very little red shift (422, 438, 554, and 605 nm) of its maximum absorption compared to the congeners. These results are also accordant with the fact that there is a minimal perturbation to no interaction of the electronic structures of the discrete macrocyclic *π*-systems in these triads. The ground state data indicate that there exists a symmetric but non-parallel arrangement of the two free or metaled axial porphyrins with respect to the plane of the central Sn(IV) porphyrin. We did not observe any kind of couplings between the porphyrin rings either axial-basal, axial-axial, or basal-basal. However, the spectrum obtained for triad **4** is different from the rest. The spectrum is broad at the Soret band region and the *λ*_max_ value (455 nm) is red-shifted compare to the corresponding monomeric units. On the other hand, the *λ*_max_ values at the Q-band appeared at 572 and 614 nm. Red shift and broad Soret band indicate that the self-assembly process occurs in a *J*-type or step-like arrangement in solution [32].

**Figure 2:**
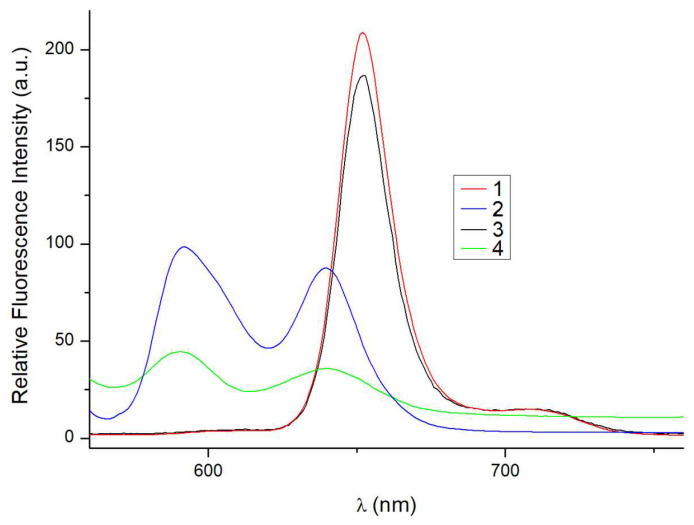
Fluorescence spectra of triads **1**–**4** in CHCl_3_. Optical density (OD) of the sample solution was fixed at 0.15.

Steady-state fluorescence spectra of all four triads were studied in CHCl_3_ and are illustrated in Figure 2. When excited at 550 nm, triads **1** and **3** showed typical two-banded fluorescence spectra, with the emission maxima (*λ*_max_) appearing at 650 and 720 nm. It was concluded that the singlet-state properties of both **1** and **3** are close to each other. The spectral pattern is very much similar to that of the free porphyrin monomer H_2_L^1^. It is likely that the influence of H_2_L^1^ is dominating over the central Sn(IV) porphyrin.

As seen in Figure 2, the emission spectra of **2** and **4** are similar and only different in intensity. The effective aggregation in **4**, due to intramolecular metal–ligand coordination, leads to the quenching of emission intensity compared to **2**. Comparing those of **1** and **3**, the emissions for **2** and **4** are considerably different due to the presence of Zn(II) in the axial porphyrins. The peak position was also blue-shifted and it appeared at 590 and 640 nm, respectively.

### 3.3. Supramolecular Self-Assembly to Nanostructures

**Figure 3:**
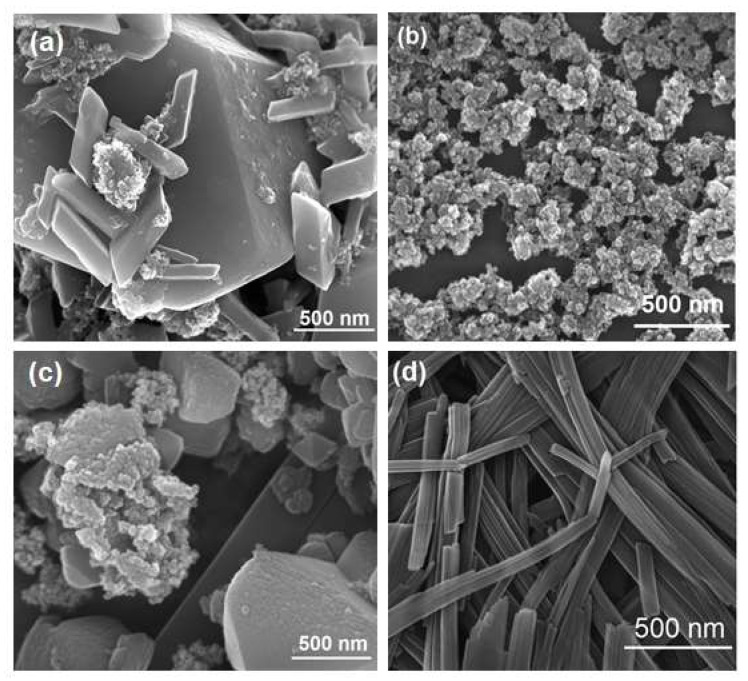
FE-SEM images for the assembly patterns of triads: (**a**) **1**; (**b**) **2**; (**c**) **3**; (**d**) **4**.

The self-assembly nature of the synthesized triads **1–4** was examined through SEM studies. For preparation of the nanostructure samples, the corresponding compounds were suspended in *n*-hexane/toluene at a fixed concentration at 1 mM and were centrifuged at 5000 rpm. During the SEM studies, drop-casting was used for the deposition of the samples on copper tapes, followed by drying. Pt coating was used before the SEM studies. All four triads assembled into nanostructures and their different morphologies are illustrated in Figure 3 and Figure S13.

A mixture of nanostructures was observed for triad **1** (Figure 3a). It consists of regular octahedra along with well-shaped parallelepiped hexahedron assemblies. The dimensions of the octahedron assemblies are in the micrometer range. On the other hand, the size of parallelepiped hexahedron assemblies was varied from nanometers to a few micrometers. These irregular morphologies were transformed into regular micro-aggregates when the free porphyrin in triad **1** was converted to Zn(II) porphyrin in triad **2**. Although, the shape of these assembled nanostructures has no distinctive features (Figure 3b). The average diameter is ranging from 150 to 500 nm. This value consists of the data obtained from the particle size analysis experiment (DLS experiments in Figure S14). A mixture of assemblies was observed for triad **3**. It consists of several mixtures of nanostructures, varying from rods to flakes and particles (Figure 3c and Figure S14c). When triad **3** was metallated, it formed triad **4**. Well-defined nanofibers were observed for triad **4** (Figure 3d) with narrow size distributions in both diameter and length. The average length of the nanofibers varied from several nanometers to a few micrometers. The average width of the nanofibers ranges from 10 to 22 nm. It should be noted that the starting monomer did not show any kind of nanostructures under our experimental conditions (Figure S15).

After analyzing the SEM images, it was clear that only triad **4** could produce a well-shaped and regular nanostructure compared to others. Now, we investigate the reason behind this type of aggregates among these triads. The porphyrin molecules aggregated via the self-assembly processes. Hydrogen bonding, ligand coordination, hydrophobic interactions, *π*-*π* stacking, and van der Waals forces are among the reason for this type of interaction. Here, all the triads formed nanostructures via the *π*-*π* stacking interaction between the adjacent porphyrin molecules through either face-to-face or edge-type interactions. However, in the case of triad **4**, the pyridyl groups in the Sn(IV) porphyrin can coordinate to the Zn ion in the axial porphyrin. It locks the movement of the axial porphyrins, and that leads to the formation of regular nano-aggregates. This type of directional self-assembly is absent from the other triads. During ^1^H NMR studies, it was observed that triad **4** was sparingly soluble in CDCl_3_. We added some pyridine-d_5_ to increase the solubility in CDCl_3_. This is because the pyridyl compounds can easily coordinate with zinc porphyrins and we can use it as an alternative guest. We used pyrrolidine instead of pyridine (to avoid the toxicity of pyridine) to investigate self-assembly studies with triad **4**. The coordination between triad **4** and pyrrolidine nitrogen was confirmed by UV-vis, fluorescence spectroscopy, and SEM studies. The fabrication nature of triad **4** was diminished after the addition of two equivalents of pyrrolidine. Absorption of the broad peak at 453 nm, which is designated to the Soret band, of the porphyrin aggregates blue-shifted to a sharp peak at 435 nm after triad 4 was coordinated by the pyrrolidine, while the peaks at 573 and 615 nm remained unchanged (Figure S16). Furthermore, an increase in the fluorescence intensity at 590 or 640 nm and a slight red shift (609 or 657 nm) of this emission peak was observed after the addition of pyrrolidine to triad 4 (Figure S17). Based on fluorescence titration experiments, the association constants *K* for the pyrrolidine binding (2:1) to triad **4** in CHCl_3_ was measured to be 2.81 × 10^4^ M^−2^. The self-assembly fabrication completely collapsed after the addition of pyrrolidine to triad **4**. The change in the morphology was confirmed by SEM images (Figure S18).

### 3.4. Photocatalysis for the Degradation of Methylene Blue (MB) Dye

**Figure 4:**
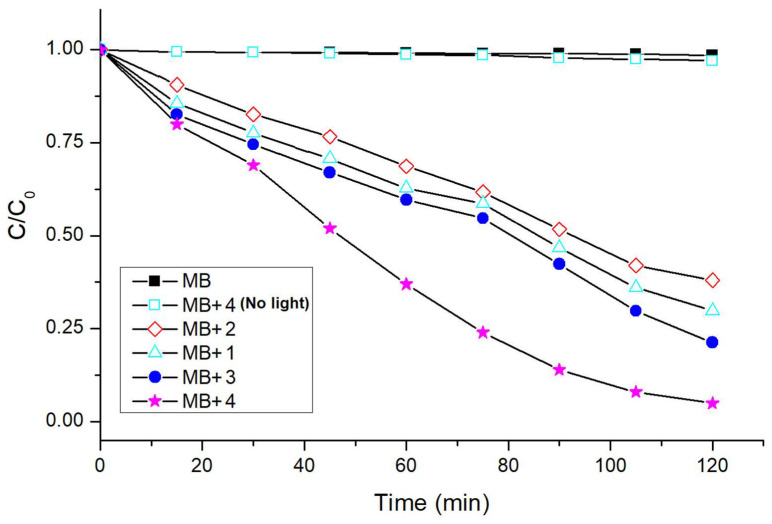
Photocatalytic degradation of methylene blue (MB) under visible light irradiation by different morphologies of Sn(IV) porphyrin-based nano-aggregates.

The photocatalytic degradation of MB dye was used to assess the photocatalytic activity of Sn(IV) porphyrin-based nano-aggregates under visible light irradiation [51,52,53,54]. A negligible decomposition of MB dye is noticed in the absence of either any photo-catalyst or visible light irradiation. Thus, all these porphyrin aggregates display photocatalytic properties under visible light irradiation only. The adsorption–desorption equilibrium between the photocatalyst and MB was reached after stirring in the dark for about 30 min. The formation of new peaks in the mass spectra compared to MB was observed after degradation studied with a photocatalyst for 2 h. These peaks, which corresponded to some small molecules, confirm the successful degradation of MB by the photocatalyst. Figure S19 shows the time-dependent absorption spectra of the aqueous solution of MB in the presence of nanofibers derived from triad **4** under light irradiation. The fall-off in the absorption peak (664 nm) of dye with time stipulates the photocatalytic degradation of MB. Figure 4 shows the *C*/*C*_0_ versus time plot of MB in the presence of divergent morphologies of Sn(IV) porphyrin-based nanostructures. It should be noted that the starting monomer did not show any catalytic performance for the degradation of MB dyes under the current experimental conditions.

It was confirmed that all the photocatalysts showed significant progress in the photodegradation of MB. These observations pronounce that the photocatalytic performance depends upon the morphology of these aggregated structures. The degradation of MB dye can be specified by its degradation efficiency, (*C*_0_−*C*)/*C*_0_, where *C*_0_ is the initial concentration of MB and *C* is the concentration at time *t*. The observed degradation rates of MB are 70%, 62%, 79%, and 95% for the nanostructures derived from triads **1**, **2**, **3**, and **4,** respectively. The photocatalytic degradation efficiency of methylene blue under UV light irradiation follows the order **4** > **3** > **1** > **2** > either no catalyst or no light. Therefore, the nanofiber aggregates derived from triad **4** shows the best photocatalytic performance toward the degradation of methylene blue.

To further elucidate the reaction kinetics for the decomposition of methylene blue, we used the pseudo-first-order model as expressed by equation ln(*C*_0_/*C*) = *k*t, which is normally used for photocatalytic degradation operation if the initial concentration of pollutant is low, where *k* is the pseudo-first-order rate constant. Figure S20 depicts the photocatalytic reaction kinetics of MB degradation based on the data plotted in Figure 4. The first-order kinetic rate constant for the decomposition of MB by triad **4** (0.028 min^−1^) is higher than that for triad **3** (0.011 min^−1^), triad **2** (0.009 min^−1^), and triad **1** (0.008 min^−1^).

These photocatalysts were stable even after degradation was studied. These catalysts were recovered by just filtering from the reaction mixture and washing with water, followed by drying in air. Powder XRD data for triad **4** confirmed the high stability of the catalyst (Figure S21). Furthermore, the efficiency of the catalyst was intact even after applying it for degradation of MB five times. Figure S22 shows the high efficiency of the photocatalyst of triad **4** for the decomposition of MB. From these observations, it is indicated that these photocatalysts, particularly triad **4**, have excellent stability and recyclability.

A possible mechanism has been given to explain the high efficiency of the decomposition of MB by nanofiber aggregates. When a nanofiber is irradiated by visible light, electrons from the valence band of triad molecules are promoted to the conduction band by crossing the bandgap. This leads to production of electron–hole pairs (*e*^−^/*h*^+^) pairs. In the case of *J*-aggregation in nanofibers, a strong intermolecular π electronic coupling occurs between the adjacent triad molecules. This can enhance reasonable electronic delocalization over the nano-aggregated molecules due to the robust intermolecular *π*–*π* interactions. The generated electron in the conduction band may move without restriction to the aggregated molecules; consequently, the generated *e*^−^/*h*^+^ pairs are effectively separated and their recombination energy is remarkably minimized. Active species, such as hydroxy radical (OH), generated by the reaction between photo-generated holes with H_2_O or OH^−^ oxidize the MB molecules to degraded products on the surface of nanofibers [55,56]. We have examined the photodegradation of MB in the presence of coumarin as a radical scavenger. The degradation of MB drastically decreased compared to when in the absence of coumarin. It implies that some portion of OH radicals was consumed by coumarin to form 7-hydroxycoumarin and the rest reacted with MB for degradation. Although we are unable to exclude other possible mechanisms with singlet oxygen or superoxide species, this preliminary investigation probably supports a pathway with OH radical species.

## 4. Conclusions

In summary, two Sn(IV) porphyrin-based triads and an analogous Zn-coordinated derivative were synthesized by using the axial bonding approach. Depending on the substituent on the porphyrin moieties, these triads self-assembled into different nanostructures. The presence and absence of metal–ligand coordination is the key factor for the formation of these nanoaggregates. These nano aggregates differed from each other not only in their morphologies but also in their photophysical properties. These assembled structures, specifically the nanofiber derived from triad **4**, manifest efficient photocatalytic performance (95%) under visible light irradiation within 2 h. Cooperative coordination between 3-pyridyl nitrogen in the Sn(IV) porphyrin with the axial Zn(II) porphyrin was responsible for the formation of uniform nanofiber aggregates. Intramolecular coordination between metal and ligands directed the self-assembly fabrication of nano-aggregates in triad **4**. This type of metal–ligand bonding was absent from other triads, leading to irregular aggregation. The self-assembly morphologies of nanofibers changed drastically upon the addition of pyrrolidine. It suggests that the coordination of pyrrolidine with axial Zn(II) porphyrin breaks down the self-assembly process in triad **4**. To the best of our knowledge, this is a novel example where intramolecular coordination between complementary porphyrins influences the morphology of the porphyrin-based nanostructures. Our observation revealed that the morphology of these nanostructures depends on the substituents mainly in the triads. Therefore, reproducibility for each and every nanostructure is easily attainable and it is the main achievement of this report. The present study presents a new strategy to construct new porphyrin-based nanostructures by using this approach and it could pave the way for elaborating new smart materials.

## Supplementary Materials

The following are available online at https://www.mdpi.com/2079-4991/10/11/2314/s1. Figure S1. ^1^H NMR spectrum of **H_2_L^1^**. Figure S2. ^1^H NMR spectrum of **ZnL^1^**. Figure S3. ^1^H NMR spectrum of **H_2_L^2^**. Figure S4. ^1^H NMR spectrum of **SnL^2^**. Figure S5. ^1^H NMR spectrum of triad **1**. Figure S6. ^1^H NMR spectrum of triad **2**. Figure S7. ^1^H NMR spectrum of triad **3**. Figure S8. ^1^H NMR spectrum of triad **4**. Figure S9. ESI-MS spectrum of triad **1**. Figure S10. ESI-MS spectrum of triad **2**. Figure S11. ESI-MS spectrum of triad **3**. Figure S12. ESI-MS spectrum of triad **4**. Figure S13. SEM images for the aggregates of **1**-**4**. Figure S14. Particle analyzing data for triad **2**. Figure S15. SEM images of the aggregates of the monomers. Figure S16. Absorption spectral changes of triad **4** upon the addition of pyrrolidine. Figure S17. Fluorescence intensity changes of triad **4** upon the addition of pyrrolidine. Figure S18. SEM image for the aggregates of triad **4** upon the addition of pyrrolidine. Figure S19. Absorption spectra of MB in presence of nano fibers derived from triad **4** under visible light irradiation. Figure S20. Kinetics for the photocatalytic degradation of MB under visible light irradiation of the four triads. Figure S21. PXRD data of triad **4** before and after an experiment on the photo-degradation of MB. Figure S22. Catalytic cycles using triad **4** as a photocatalyst for the degradation of MB.
