# Supplementary material for: Supramolecular Porphyrin Nanostructures Based on Coordination-Driven Self-Assembly and Their Visible Light Catalytic Degradation of Methylene Blue Dye
Source: Nanomaterials (Basel). 2020 Nov 22;10(11):2314. doi: 10.3390/nano10112314 (PMC7700138; doi:10.3390/nano10112314)
Supplement: Supplementary file 1 [file nanomaterials-10-02314-s001.pdf]

nanomaterials

Supplementary Materials

Supramolecular Porphyrin Nanostructures Based on Coordination-Driven Self-Assembly and Their Visible Light Catalytic Degradation of Methylene Blue Dye

Nirmal Kumar Shee, Min Kyoung Kim and Hee-Joon Kim *

Department of Applied Chemistry, Kumoh National Institute of Technology, Gumi 39177, Korea; nirmalshee@gmail.com (N.K.S.); min1107min@naver.com (M.K.K.)

* Correspondence: hjk@kumoh.ac.kr; Tel.: +82-(54)-4787822

List of contents:

Figure S1. ^{1}H NMR spectrum of *meso*-5-(4-hydroxyphenyl)-10,15,20-tris(3,5-di-*tert*-butylphenyl)porphyrin in $CDCl_3$.

Figure S2. ^{1}H NMR spectrum of *meso*-(5-(4-hydroxyphenyl)-10,15,20-tris(3,5-di-*tert*-butylphenyl)porphyrinato) zinc(II) in $CDCl_3$.

Figure S3. ^{1}H NMR spectrum of 5,15-bis(3-pyridyl)-10,20-bis(phenyl)porphyrin in $CDCl_3$.

Figure S4. ^{1}H NMR spectrum of *trans*-dihydroxo-(5,10,15,20-tetrakis(3-pyridyl)porphyrinato) tin(IV) in $CDCl_3$.

Figure S5. ^{1}H NMR spectrum of triad **1** in $CDCl_3$.

Figure S6. ^{1}H NMR spectrum of triad **2** in $CDCl_3$.

Figure S7. ^{1}H NMR spectrum of triad **3** in $CDCl_3$.

Figure S8. ^{1}H NMR spectrum of triad **4** (2 mM) in $CDCl_3$.

Figure S9. ESI-MS spectrum of triad **1**.

Figure S10. ESI-MS spectrum of triad **2**.

Figure S11. ESI-MS spectrum of triad **3**.

Figure S12. ESI-MS spectrum of triad **4**.

Figure S13. SEM images for the aggregates of triads: (**a**) **1**; (**b**) **2**; (**c**) **3**; (**d**) **4**.

Figure S14. Particle analyzing data for triad **2** in a *n*-hexane solution.

Figure S15. SEM images of the aggregates of the monomers: (a) ZnL^1; (b) SnL^2.

Figure S16. Absorption spectral changes of triad **4** upon the addition of pyrrolidine in $CHCl_3$.

Figure S17. Fluorescence intensity changes of triad **4** upon the addition of pyrrolidine in $CHCl_3$.

Figure S18. SEM image for the aggregates of triad **4** upon the addition of pyrrolidine.

Figure S19. Absorption spectra of MB in presence of nano fibers derived from triad **4** under visible light irradiation.

Figure S20. Kinetics for the photocatalytic degradation of MB under visible light irradiation of the four triads (**1**, **2**, **3**, and **4**).

Figure S21. PXRD data of triad **4** before and after an experiment on the photo-degradation of MB.

Figure S22. Catalytic cycles using triad **4** as a photocatalyst for the degradation of MB.

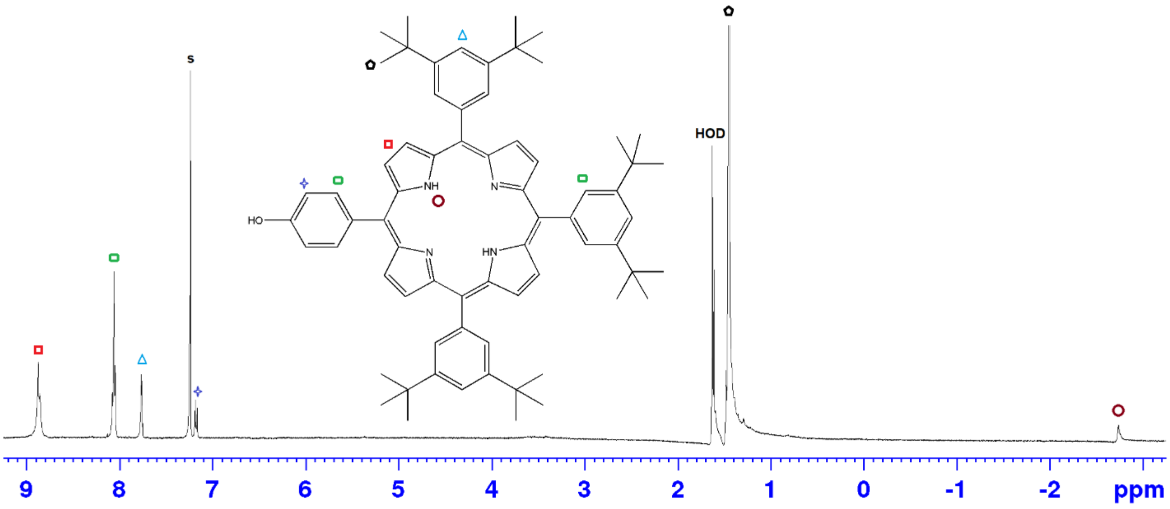

Figure S1. ^{1}H NMR spectrum of *meso*-5-(4-hydroxyphenyl)-10,15,20-tris(3,5-di-*tert*-butylphenyl)porphyrin in $CDCl_3$.

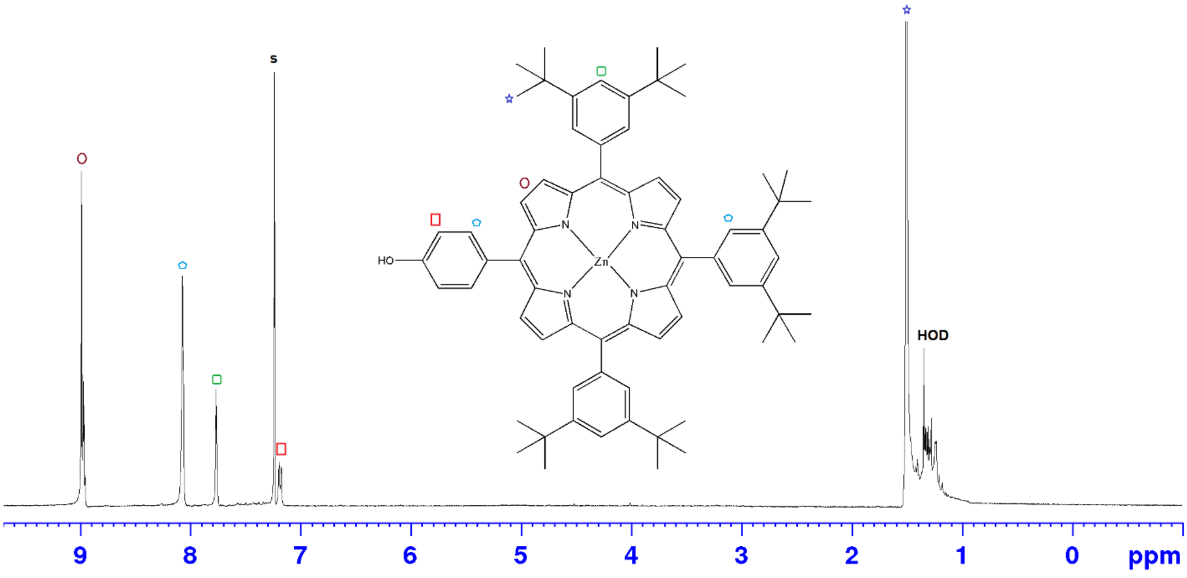

Figure S2. ^{1}H NMR spectrum of *meso*-(5-(4-hydroxyphenyl)-10,15,20-tris(3,5-di-*tert*-butylphenyl)porphyrinato) zinc(II) in $CDCl_3$.

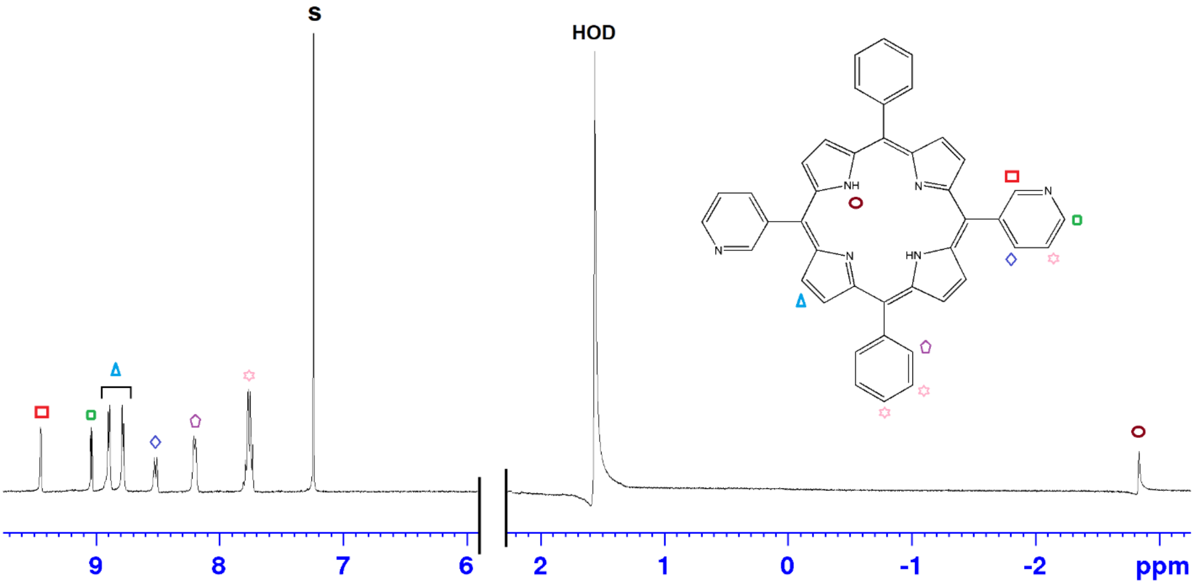

Figure S3. ^{1}H NMR spectrum of 5,15-bis(3-pyridyl)-10,20-bis(phenyl)porphyrin in $CDCl_3$.

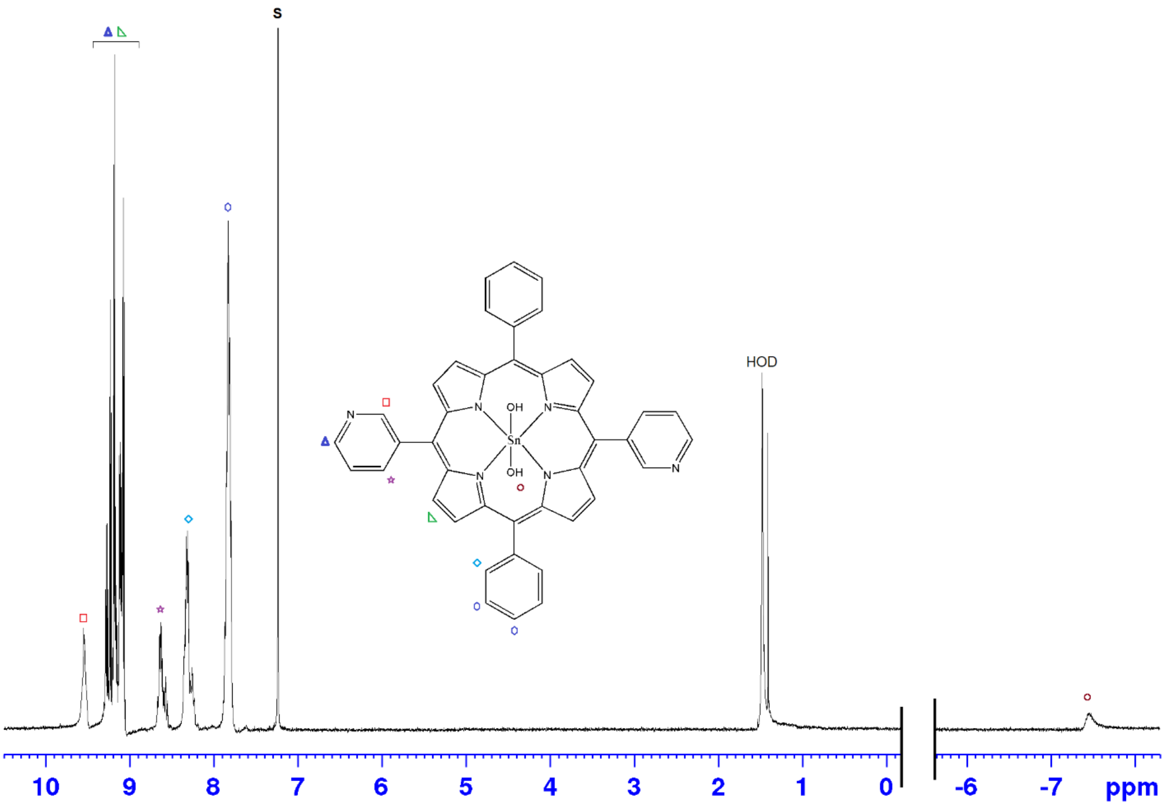

Figure S4. ^{1}H NMR spectrum of *trans*-dihydroxo-(5,10,15,20-tetrakis(3-pyridyl)porphyrinato) tin(IV) in $CDCl_3$.

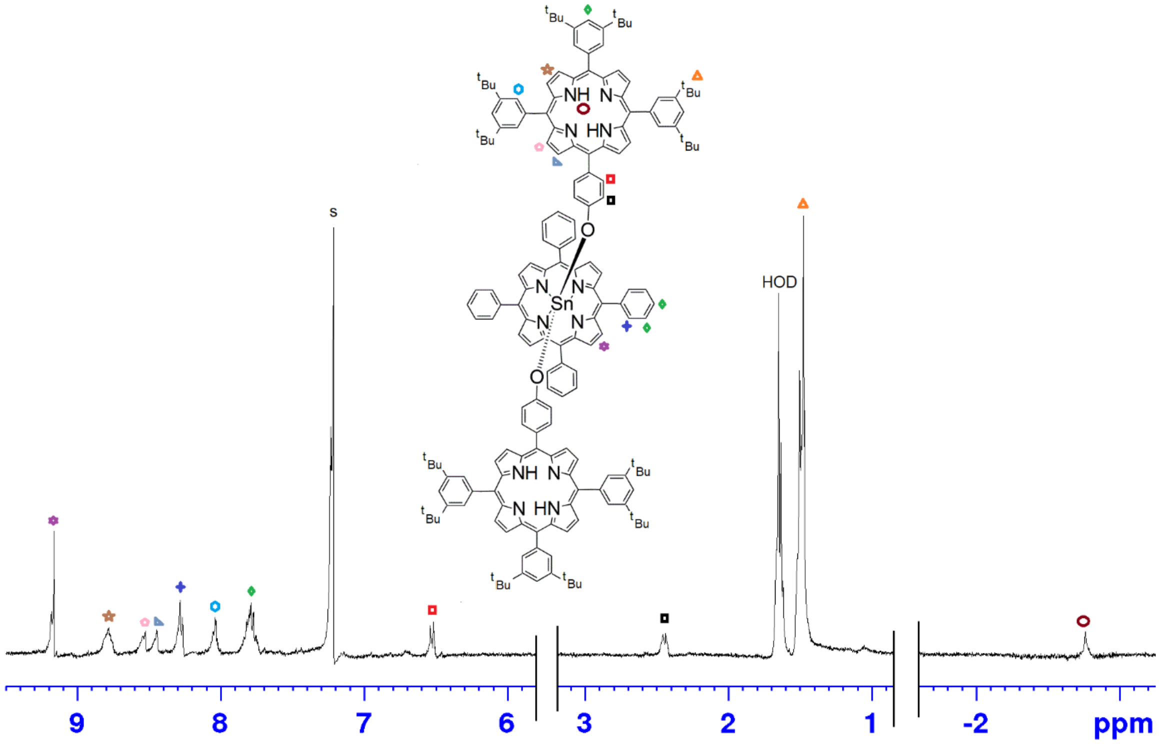

Figure S5. ^{1}H NMR spectrum of triad **1** in $CDCl_3$.

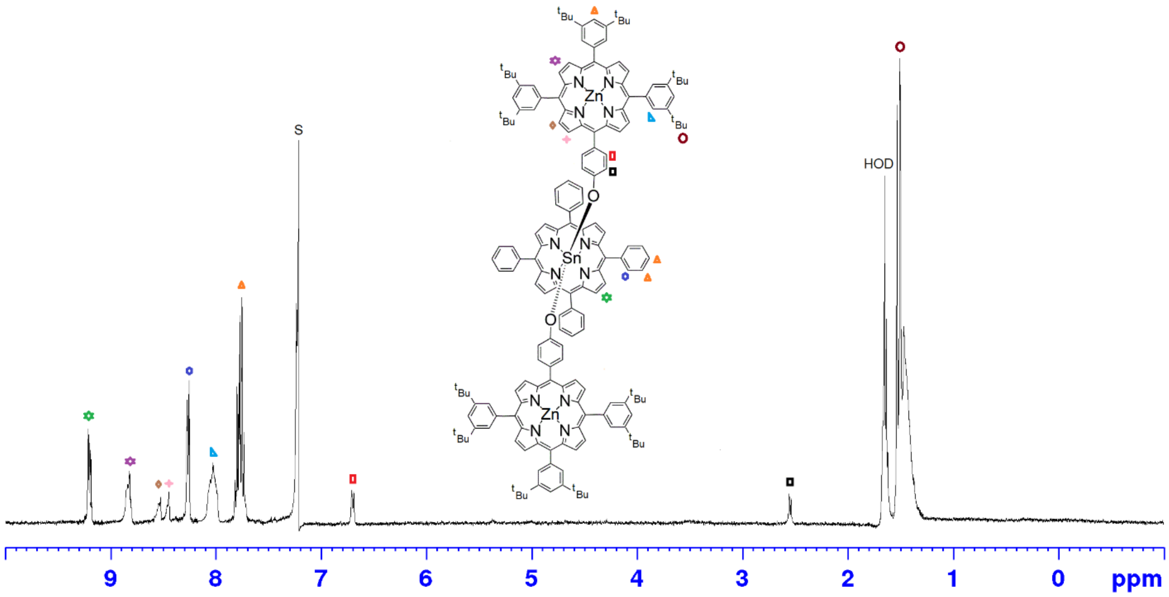

Figure S6. ^{1}H NMR spectrum of triad **2** in $CDCl_3$.

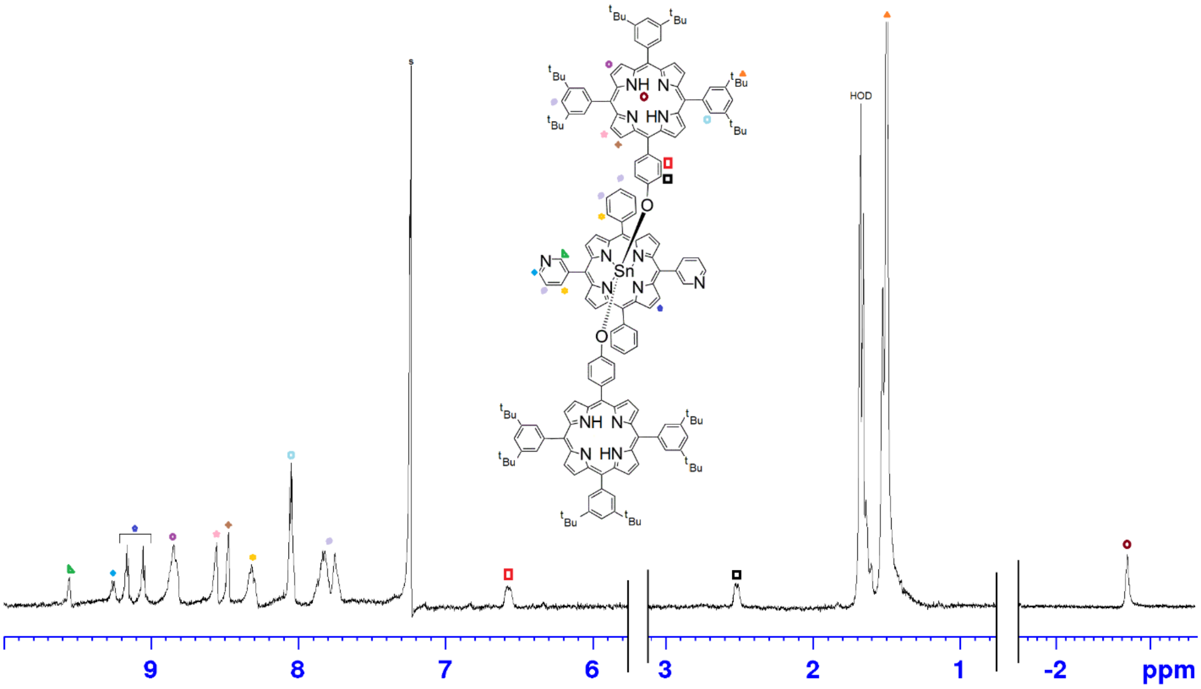

Figure S7. ^{1}H NMR spectrum of triad **3** in $CDCl_3$.

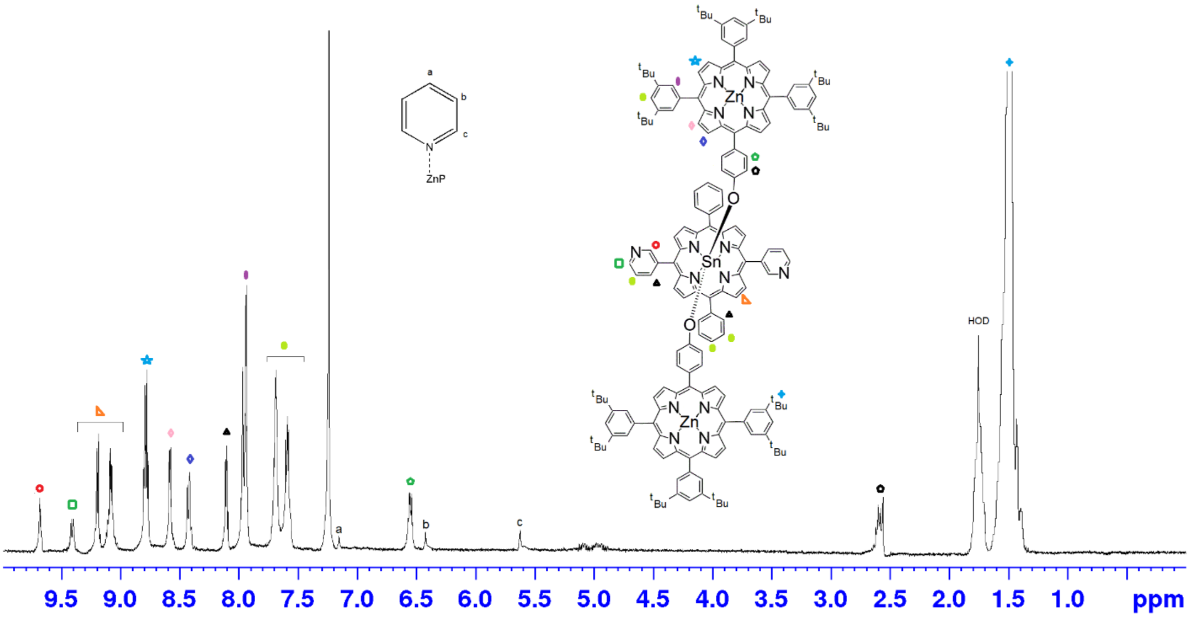

Figure S8. ^{1}H NMR spectrum of triad **4** (2 mM) in $CDCl_3$ after adding 2 eq. pyridine-d_5 (as the solubility of triad **4** in $CDCl_3$ is very low). In the presence of bases such as pyridine, triad **4** becomes much soluble in $CDCl_3$. Residual solvent peaks of pyridine coordinated with Zn porphyrin were assigned as a, b, and c respectively.

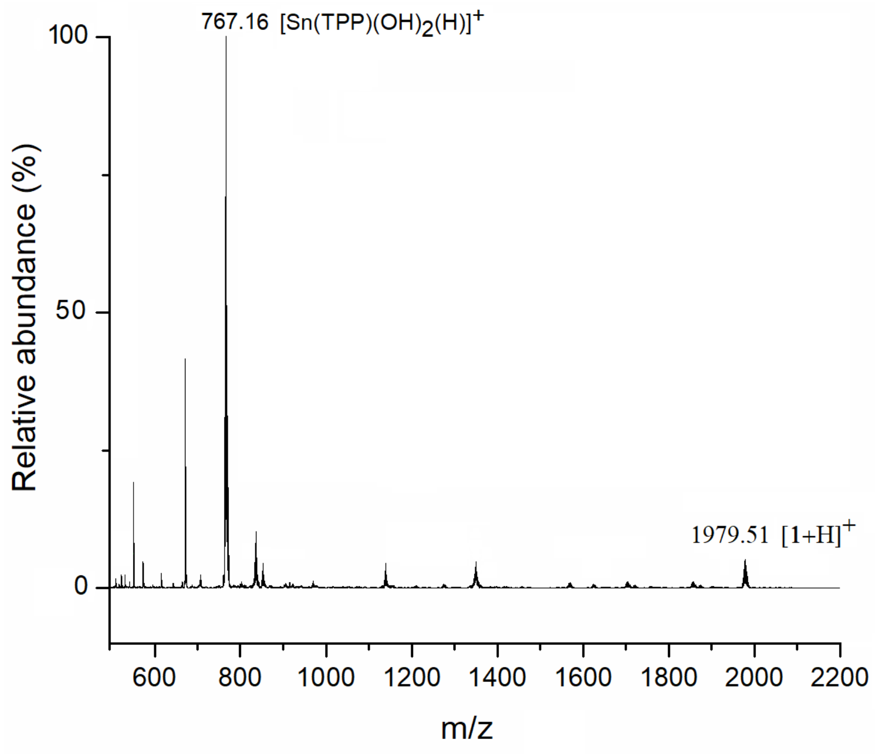

Figure S9. ESI-MS spectrum of triad **1**.

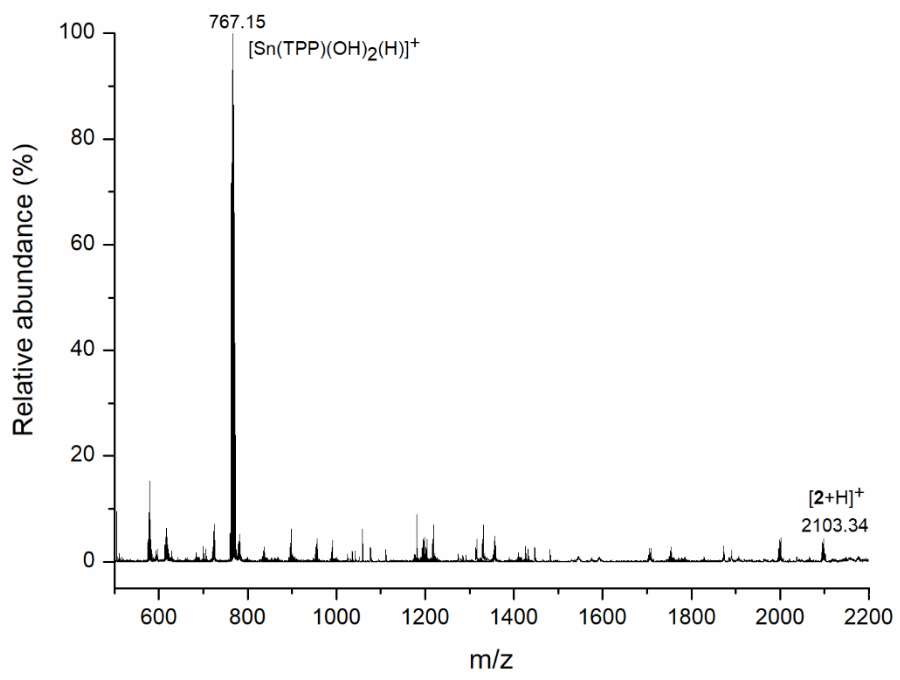

Figure S10. ESI-MS spectrum of triad **2**.

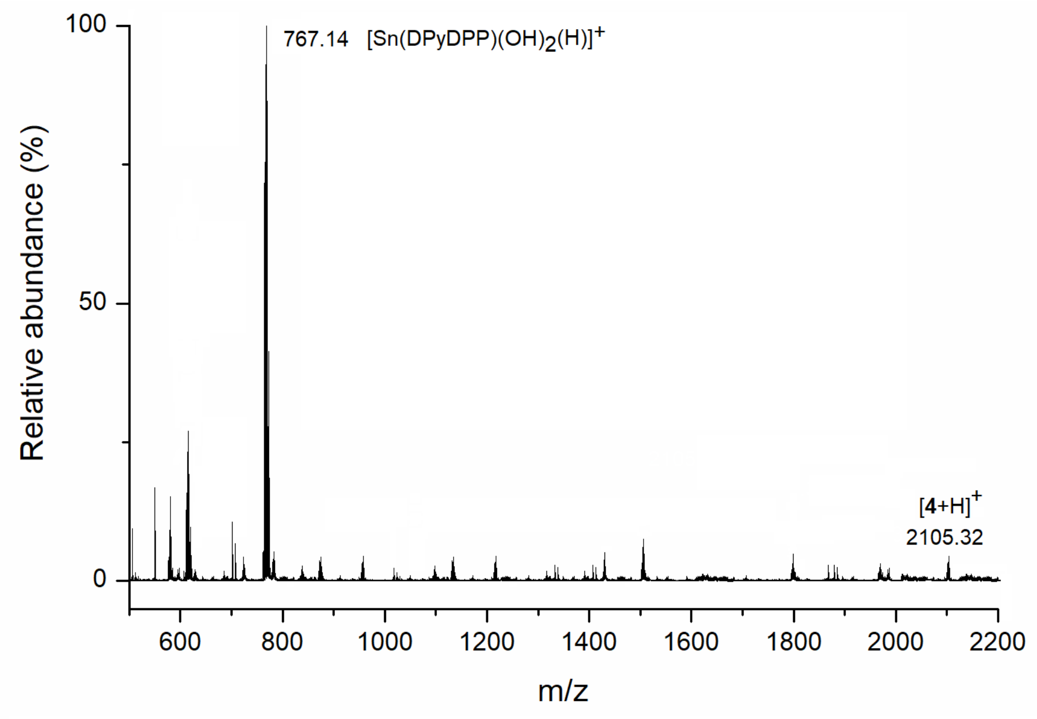

Figure S11. ESI-MS spectrum of triad **3**.

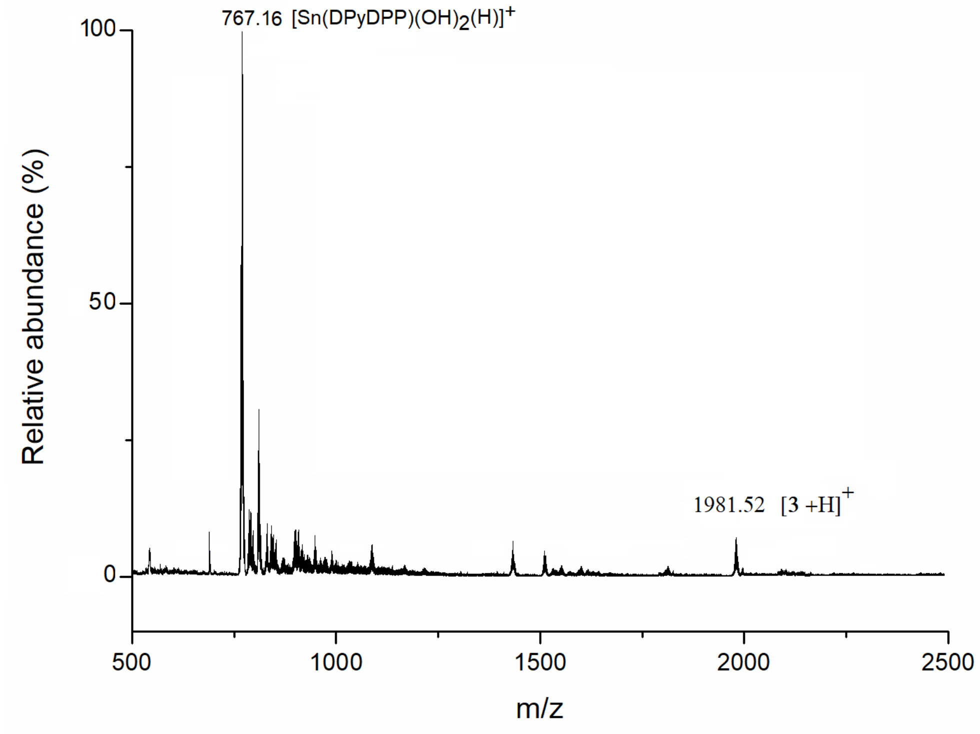

Figure S12. ESI-MS spectrum of triad **4**.

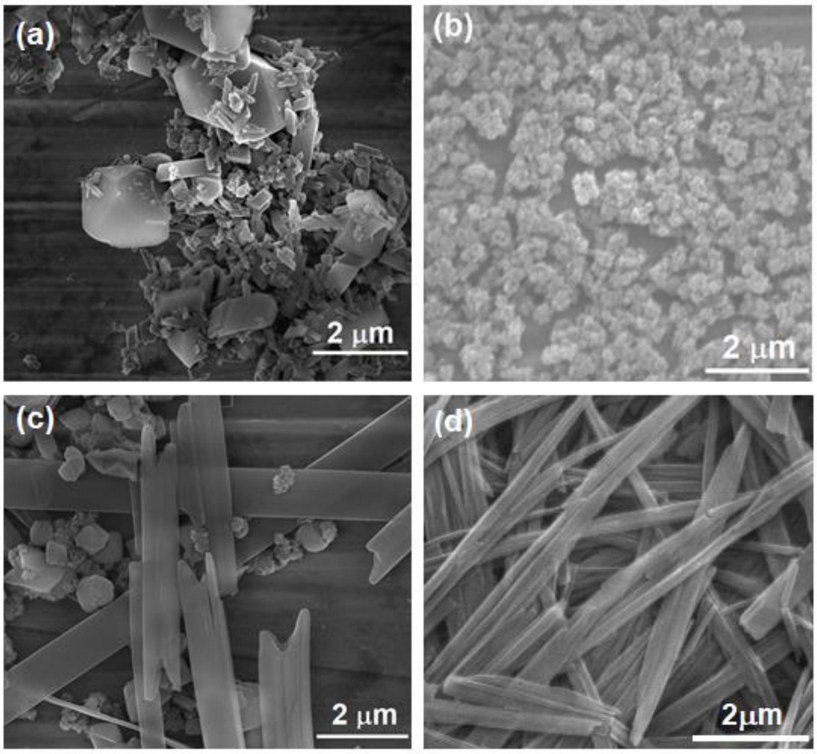

Figure S13. SEM images for the aggregates of triads: (**a**) **1**; (**b**) **2**; (**c**) **3**; (**d**) **4**.

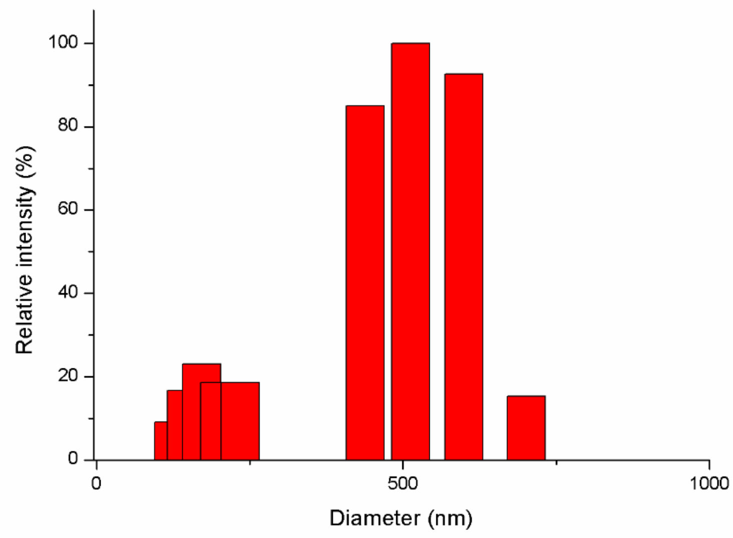

Figure S14. Particle analyzing data for triad **2** in a *n*-hexane solution.

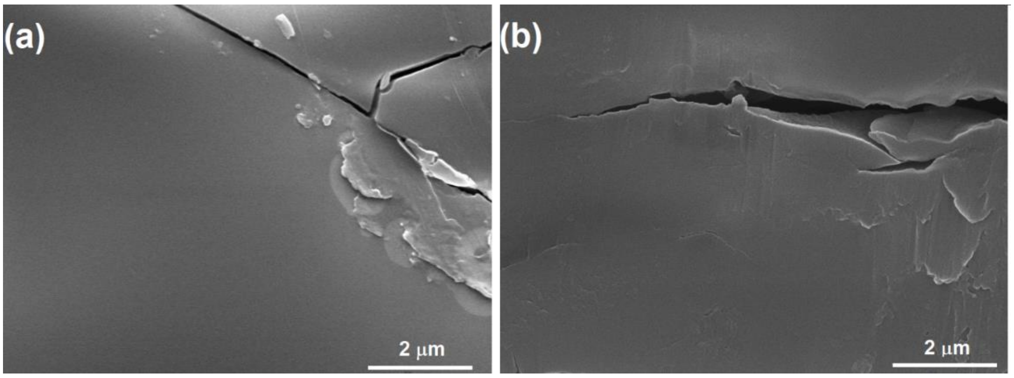

Figure S15. SEM images for the aggregates of the monomers: (**a**) **ZnL**[1]; (**b**) **SnL**[2].

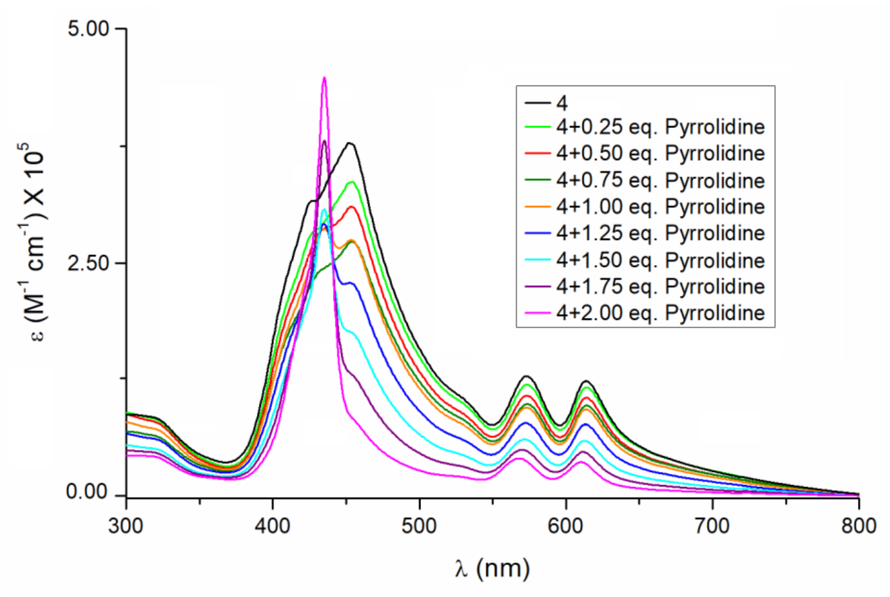

Figure S16. Absorption spectral changes of triad **4** upon the addition of pyrrolidine in $CHCl_3$.

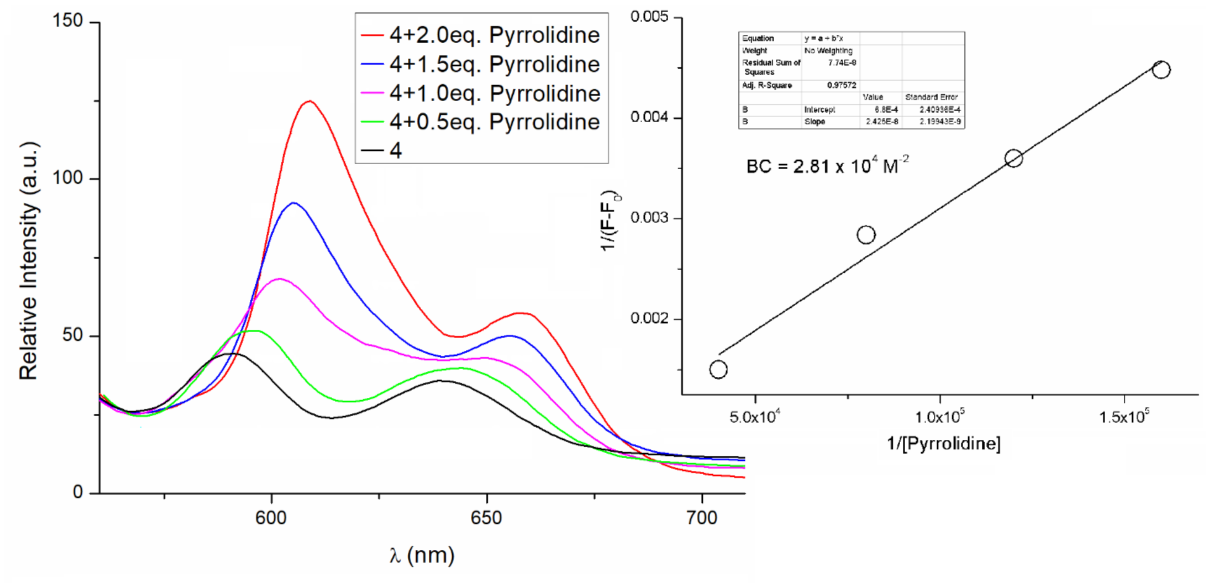

Figure S17. Fluorescence intensity changes of triad **4** upon the addition of pyrrolidine in $CHCl_3$.

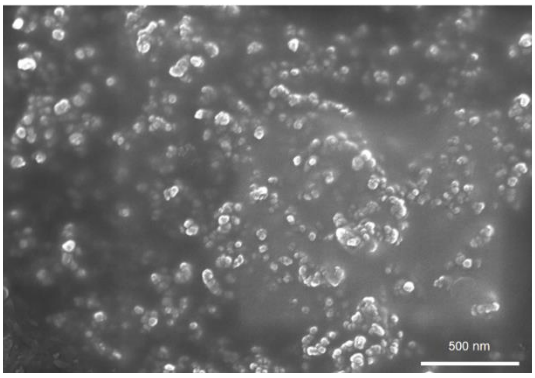

Figure S18. SEM image for the aggregates of triad **4** upon the addition of pyrrolidine.

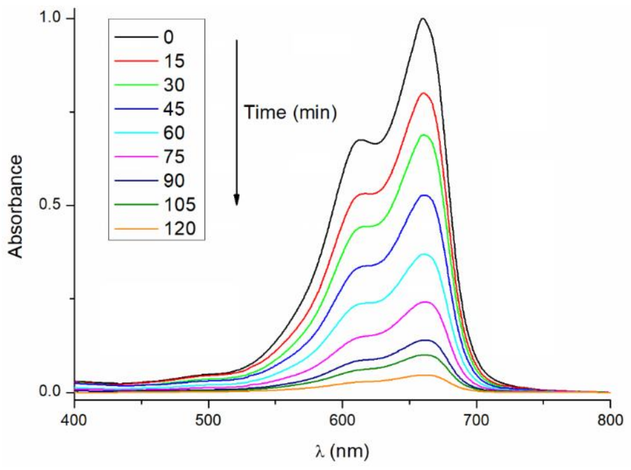

Figure S19. Absorption spectra of MB in presence of nano fibers derived from triad **4** under visible light irradiation.

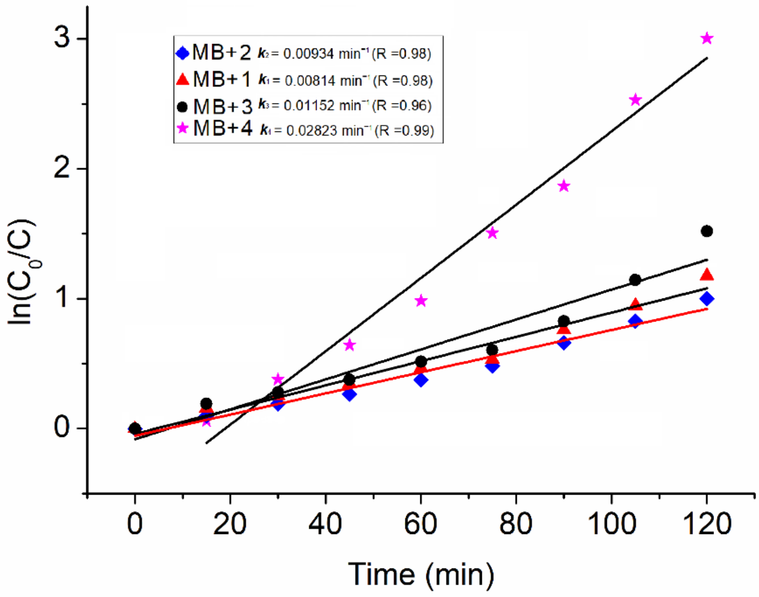

Figure S20. Kinetics for the photocatalytic degradation of MB under visible light irradiation of the four triads (**1**, **2**, **3**, and **4**).

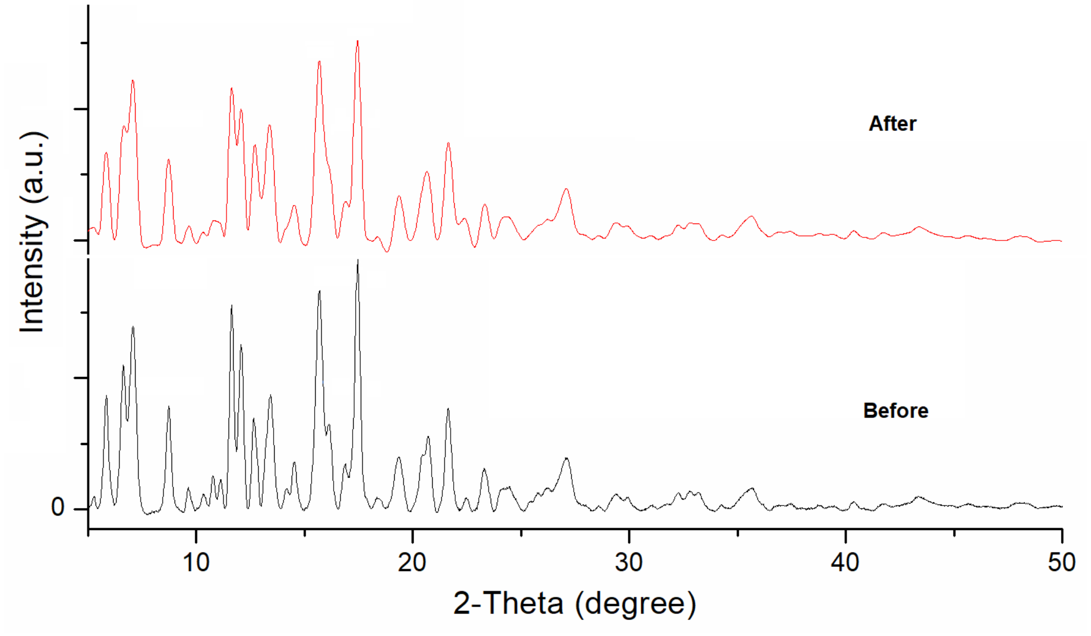

Figure S21. PXRD data of triad **4** before and after an experiment on the photo-degradation of MB.

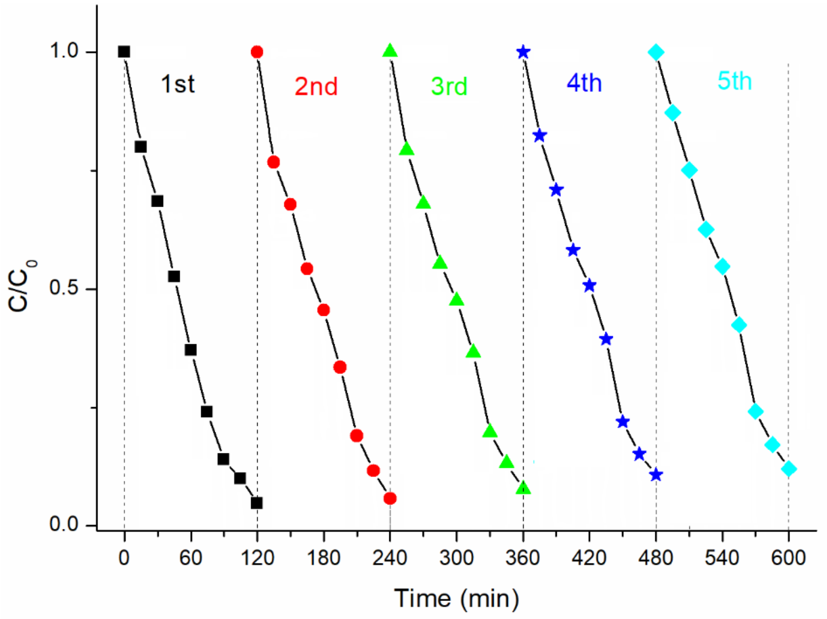

Figure S22. Catalytic cycles using triad **4** as a photocatalyst for the degradation of MB.
